# Clinical manifestations associated with the chronic phase of Chikungunya Fever: A systematic review of prevalence

**DOI:** 10.1371/journal.pntd.0012810

**Published:** 2025-02-03

**Authors:** Raphael Augusto Santiago, Suelen Pereira Priante Bavaresco, Sheyla Goulart Citrangulo, Roberto de Andrade Medronho, Vanderson Sampaio, Antônio José Leal Costa

**Affiliations:** 1 Institute of Studies in Collective Health, Federal University of Rio de Janeiro, Rio de Janeiro, State of Rio de Janeiro, Brazil; 2 Faculty of Medicine, Federal University of Amazonas, Manaus, State of Amazonas, Brazil; 3 Tropical Medicine Foundation Dr. Heitor Vieira Dourado, Manaus, State of Amazonas, Brazil; Public Health Agency of Canada, CANADA

## Abstract

**Introduction:**

The aim of this systematic review of prevalence is to observe and discuss the clinical manifestations of Chikungunya Virus disease in its chronic phase.

**Methods:**

To be eligible, the observational studies should accompany the individuals for at least six months. The research was conducted using electronic databases MEDLINE and EMBASE. The methodological quality was evaluated using the “*Joanna Briggs Institute’s critical appraisal checklist for studies reporting prevalence data*” tool.

**Results:**

The search has found 175 articles. The application of the inclusion criteria defined a total of 29 selected studies. From the included studies, only one did not present arthralgia as a prevalent symptom in the chronic phase. Other signs and symptoms observed were: fatigue; sleep disorders; myalgia; skin lesions; depression; digestive disorders.

**Conclusion:**

Because it is an often incapacitating symptom, arthralgia can affect the individuals’ quality of life, with implications in their social and work life. Since the chronic phase is common in infected individuals, all levels of health care should be prepared to monitor, in the medium to long term, the patients affected by this condition.

## Synopsis

The knowledge acquired regarding the high prevalence of several arboviruses present in the world, some of which spread rapidly to various regions of the planet, generating coexistence and coinfections, demonstrates the importance of a better understanding of the impact that each of them has on the health of infected individuals. In this scenario, differential diagnosis becomes an essential factor for the correct treatment of the signs and symptoms presented. Therefore, with this study we sought to expand knowledge about the epidemiology and clinical manifestations present in arboviruses, highlighting the Chikungunya Virus as one of the most prevalent and symptomatic arboviruses.

The results of this study aim to expand the epidemiological surveillance of Chikungunya fever by contributing to the better identification of clinical signs and symptoms, identifying its chronic phase and assisting in the differential diagnosis for other arboviruses with similar performance, such as infection by the Dengue and Zika viruses.

## Introduction

The Chikungunya Fever (CHIKF) is an infection caused by the Chikungunya Virus (CHIKV), an arbovirus of the *Togaviridae* family, of the genus *Alphavirus*, whose viremia persists for up to ten days after the onset of signs and symptoms [1]. The CHIKV was first isolated in Tanzania around 1952, and, since then, outbreaks have been observed in several countries [1,2,3]. In 2013, CHIKV was inserted in the Caribbean, expanding itself in 2014 to the continental areas of the Americas [4,5].

The main route of transmission is vectorial, which occurs due to the bite of the *Aedes aegypti* and the *Aedes albopictus* female mosquitoes–whose infestation rates increase with summer rains–infected by CHIKV [1,4,6]. The virus’ incubation period in human beings is on average three to seven days, ranging from one to twelve days [1,7].

Approximately 70% of the subjects infected with CHIKV show signs and symptoms; a high proportion when compared to other arboviruses [1]. Several clinical manifestations can be observed in patients with CHIKF. The virus ability to reproduce itself in different body tissues, from the integument to the central nervous system, through the heart muscle, the joints, the liver, and others, generates a high variability of clinical signs and symptoms [8]. The clinical manifestations are similar to those found in dengue virus infection: acute fever, arthralgia, myalgia, cephalea, nausea, fatigue and rash [1,3,7,9].

CHIKF can evolve in three stages: Acute or febrile phase; Subacute phase and Chronic phase [1,7]. The acute phase is characterized by sudden fever and high intensity arthralgia. Polyarthralgia has been described in over 90% of the cases in this phase, in which the condition is usually symmetrical—although asymmetry is possible, it affects large and small joints, and encompasses the most distal regions more frequently. May be associated with edema, which is usually related to tenosynovitis [1]. Ligament pain and myalgia, especially in the upper limbs and thighs, were also observed in the acute phase [1]. This phase lasts, on average, seven days [1,10].

In the subacute phase, arthralgia may persist or get even worse, presenting distal polyarthritis, increased pain in the regions already affected in the acute phase, and subacute hypertrophic tenosynovitis in the wrists and ankles [1,10].

After the subacute phase, some symptoms, especially arthralgia and myalgia, may persist in some patients [1,10–12]. Typically, the symptoms of CHIKF disappear within one to three weeks. However, some subjects may present a recurrence of rheumatological manifestations, such as polyarthralgia, polyarthritis and tenosynovitis, in the months after the acute condition [3,7].

Although CHIKV infection has low lethality, in many cases it progresses to the chronic phase, and it may incapacitate the patient for weeks and even for several years after the acute phase, leading to reduced movements and quality of life [11,13,14].

There are no antivirals or vaccines available for the treatment or prevention of the CHIKV infection [15]. The current treatment consists on the use of analgesics, antipyretics and anti-inflammatory agents, such as paracetamol and non-steroidal anti-inflammatory drugs [16,17].

As mentioned, joint impairment is present in most patients who develop clinical signs and symptoms of the CHIKV infection [1,11]. Therefore, a better understanding of the prevalence of characteristic signs and symptoms of the chronic phase, especially those related to joint impairment, will be able to contribute to the diagnosis and prognosis of these patients, as well as to measure the load of morbidity/disability resulting from CHIKF to provide the necessary care, including intersectoral actions.

Thus, we conducted a systematic review of prevalence, with the objective of describing the clinical manifestations of the CHIKV disease in its chronic phase.

## Methods

### Ethics statement

All studies included in this review should be approved by the respective local ethics committees. There is no protocol registered for this systematic review.

This is a systematic review of the prevalence of observational cohort studies that evaluated clinical signs and symptoms present in the chronic phase of CHIKF. The research was conducted using the electronic databases MEDLINE (accessed via PubMed) and EMBASE. The following keywords were used: *Chikungunya Virus*; *Chikungunya Fever*; *Arthralgia*, *Fever*, *Signs and Symptoms*, using Boolean operators OR and AND to associate these variables as a research strategy—((“Chikungunya Virus” OR “Chikungunya Fever”) AND (Arthralgia OR Fever OR “Signs and Symptoms”)). No limits were applied for language or year of publication.

### Condition

The variable of interest is the presentation of the chronic phase of CHIKF, defined by the permanence of the clinical manifestations’ measurement, presented in the acute phase beyond the three weeks usually expected.

### Context

In order to search for several observational studies able of describing the clinical signs and symptoms of the chronic phase of CHIKF, no limits were established for language or year of publication, avoiding to delimit geographical regions or period of time.

### Population

The inclusion criteria for the studies were: tracking the individuals in the cohort, infected by CHIKV via vector transmission and with laboratory confirmation using reverse transcription, followed by polymerase chain reaction (RT-PCR) [18,19] for at least six months; describing the clinical manifestations presented in the chronic phase of CHIKF. As exclusion criteria we have: different studies, but from the same region, that addressed the same cohort of individuals.

### Data collection and analysis

Potentially relevant articles were individually evaluated by two reviewers. A flow diagram was created, which describes the study selection process, based on the PRISMA Recommendation (Preferred Reporting Items for Systematic Reviews and Meta-Analyses) [20,21], used for this study’s design. Disagreements regarding the inclusion and exclusion of studies among the reviewers were solved through the evaluation and opinion of a third reviewer. The search for the articles was closed in October 12, 2024.

The analysis of the information from the included studies was based on: the description of the study’s design (prospective or retrospective); the number of participants included in each study, as well as the total number of individuals included in this review; the mean age; the length of each cohort’s follow-up; the presence of signs and symptoms observed in the chronic phase of CHIKF; the observation of the presence or absence of factors concomitant to the chronic phase and/or comorbidities.

### Methodological quality assessment

The risk of bias in the selected studies was assessed using the “*Joanna Briggs Institute’s critical appraisal checklist for studies reporting prevalence data*” [22], a revised tool that was specifically developed for researches that present prevalence data [22].

Each research included in this study was categorized according to the percentage of affirmative answers among the nine questions available in the evaluation tool. The risk of bias was considered high when the study presented up to 49% of the answers classified as "yes", moderate when obtained from 50% to 69%, and low when the survey exceeded 70% of "yes" scores [23].

## Results

**Fig 1:**
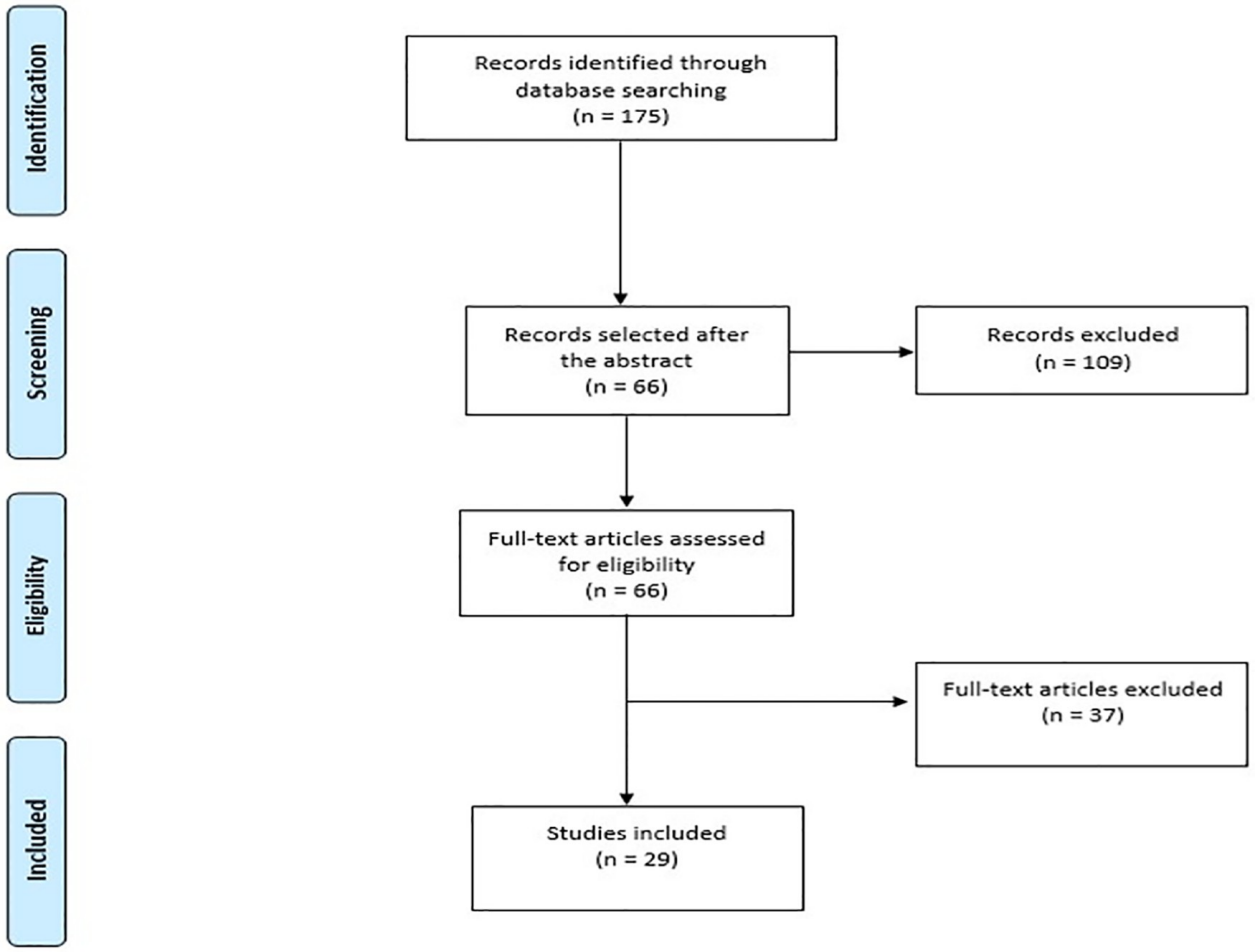
Flow chart—Study selection process.

The study selection process is presented in the flow chart in Fig 1. The search identified 175 articles, which underwent an abstract screening, and later an eligibility following the inclusion criteria, defining the process that totaled 29 studies selected to compose this review. The 109 studies that were excluded after analysis of the abstract did not present an observational design. The 37 articles excluded after analysis of the full text did not present a minimum follow-up time of six months or did not address the chronic phase of the disease.

The characteristics of the included studies are described in Table 1. Among the 29 studies, 18 presented retrospective design [12,24–40], and 11 presented prospective design [41–51]. The study with the smallest cohort of individuals used 21 subjects [41], and the largest used 5,344 subjects [40]. The total sample consists of 11,378 individuals. The mean age ranged from 32 [47] to 58.3 years [24]. The minimum follow-up time observed was of 6 months [34,47], while the longest follow-up was of 72 months [33,46].

**Table 1:** Characteristics of the *cohort observational studies included in the review.

Author/ year	Country	Design	Sample’s Size (CHIKV +)	Mean Age	Maximum follow-up time
BORGHERINI et al / 2008 [24]	Reunion Island (France)	Retrospective	88	58,3	18 months
BOUQUILLARD, COMBE / 2009 [41]	Reunion Island (France)	Prospective	21	57	17 months
SISSOKO et al / 2009 [25]	Reunion Island (France)	Retrospective	147	52	15 months
SOUMAHORO et al / 2009 [26]	Reunion Island (France)	Retrospective	199	42	17 months
LARRIEU et al / 2010 [42]	France	Prospective	29	51	24 months
MANIMUNDA et al / 2010 [43]	India	Prospective	203	35	10 months
GERARDIN et al / 2011 [27]	Reunion Island (France)	Retrospective	512	36	16 months
MATHEW et al / 2011 [28]	India	Retrospective	437	48,4	15 months
CHOPRA et al / 2012 [12]	India	Retrospective	509	NI	20 months
COUTURIER et al / 2012 [29]	France	Retrospective	391	50,2	23,4 months
MORO et al / 2012 [44]	Italy	Prospective	250	82,8% of the individuals over 40 years	13 months
ESSACKJEE et al / 2013 [30]	Mauritius Island	Retrospective	173	52,1	27,5 months
MOHD ZIM et al / 2013 [31]	Malaysia	Retrospective	53	49,2	20 months
SCHILTE et al / 2013 [45]	Reunion Island (France)	Prospective	180	NI	36 months
RAMACHANDRAN et al / 2014 [32]	India	Retrospective	403	37,7	12 months
JAVELLE et al / 2015 [33]	Reunion Island (France)	Retrospective	159	51	72 months
MARIMOUTOU et al / 2015 [46]	Reunion Island (France)	Prospective	81	44	72 months
van GENDEREN et al / 2016 [47]	Suriname	Prospective	98	32	6 months
RODRIGUEZ-MORALES et al /2016 [34]	Colombia	Retrospective	131	41	6 months
ZEANA et al / 2016 [35]	USA	Retrospective	28	51,5	9 months
BOUQUILLARD et al / 2017 [48]	Reunion Island (France)	Prospective	307	54	32 months
CONSUEGRA-RODRIGUEZ et al / 2017 [36]	Colombia	Retrospective	51	44,3	15,3 months
CHANG et al / 2018 [49]	Colombia	Prospective	494	49,1	20 months
DUVIGNAUD et al / 2018 [37]	Reunion Island (France)	Retrospective	512	30 to 59	24 months
HUITS et al /2018 [50]	Aruba	Prospective	171	49,4	18 months
MURILLO-ZAMORA et al / 2018 [38]	Mexico	Retrospective	217	41,2	12 months
PETERS et al / 2018 [39]	Saint Martin Island (Netherlands)	Retrospective	56	47	15 months
de MORAES et al /2020 [51]	Brazil	Prospective	134	39	12 months
NINLA-AESONG et al / 2020 [40]	Thailand	Retrospective	5344	NI	60 months
Total	14 countries		11378	---	---

Note–

*minimum follow-up time: 6 months;

NI: Non Informed

The results derived from signs and symptoms observed in the chronic phase of CHIKF in the studies used are shown in Table 2. Among the 29 studies included in this review, only one did not identify arthralgia as a prevalent symptom in the chronic phase of CHIKF [41]. Other signs and symptoms observed in the selected studies were: fatigue [26,27,30,37,39,44–46]; myalgia [26,35,37,38,44,45]; sleep disorders [26,27,39,45]; skin lesions [26,27,39,45]; depression [26,27,45,46]; and digestive disorders [26,27].

**Table 2:** Signs and symptoms present in the chronic phase of CHIKF in the *cohort observational studies included in the review.

Clinical signs and symptoms in the chronic phase	Number of studies/ (%)	Absolute / relative frequency (%)	
		N	%
Arthralgia	28 (96,5)	3.125	27,5
Fatigue	8 (27,6)	957	8,4
Myalgia	6 (20,7)	367	3,2
Sleep disorders	4 (13,8)	256	2,2
Skin lesions	4 (13,8)	242	2,1
Depression	4 (13,8)	135	1,2
Digestive disorders	2 (6,9)	106	0,9

Note: CHIKF–Chikungunya Fever;

*Minimum follow-up time: 6 months; Review’s total sample: 11.378.

Present in 27.5% of this review’s total sample, arthralgia is distributed as follows in the samples by study region: 52% (1243 of 2379) in Africa [24–27,30,33,37,41,45,46,48]; 46% (311 of 670) in Europe [29,42,44]; 44% (590 of 1352) in Latin America and the Caribbean [34,36,38,39,47,49–51]; 25% (7 of 28) in North America [35]; and 14% (974 of 6949) in Asia [12,28,31,32,40,43].

Concomitant factors to the chronic phase of CHIKF were observed, such as pre-existing arthritis [24,25,27,29,31,33,44], advanced age [25,29,45] and pre-existing musculoskeletal impairment [30], as well as the presence of comorbidities, such as diabetes mellitus [24,25,33,48] and arterial hypertension [25].

The results of the risk of bias analyses are shown in a table in S1 Appendix. The methodological quality of the studies included in this review was classified as follows: 20 studies (69%) presented a low risk of bias [12,24,26,27,29,31,33–35,37–41,43,44,48–51]; 5 studies (17%) a moderate risk [25,30,36,45,47]; and 4 studies (14%) a high risk [28,32,42,46].

## Discussion

The present review sought to describe the clinical manifestations during the chronic phase of the CHIKF. Among the multiple signs and symptoms shown, arthralgia was predominantly observed in 28 of the 29 observational studies included, present in approximately one quarter of the patients that make up the total sample of this review.

In addition to fever, arthralgia is the most common symptom of CHIKF, being present in practically all cases of the acute phase [17]. In other studies, the prevalence of chronic pain manifestations in the joints varied considerably, and may be presented as less than 15%, for up to almost 90% [12,24,33,43,52–54]. Such variation is due to the different methodological approaches featured, such as the study design, the selection criteria and the research scenario, for example.

Rodriguez-Morales et al. (2015) [52], analyzing eight observational studies through a nonlinear regression model, stated that 47.6% of patients with CHIKF in Latin America and the Caribbean will present chronic arthralgia. This piece of data is similar to the prevalence found in the present review in relation to Latin American countries. Our study recruited researches from fourteen countries, six of which are Latin American (Aruba [50], Brazil [51], Colombia [34,36,49], San Martin Island [39], Mexico [38] and Suriname [47]), comprising eight studies from this region. There was no evidence of predominance of the CHIKF’s clinical manifestations due to race or ethnic groups.

Although arthralgia is already recognized as the fundamental characteristic of CHIKF, there is a small number of studies addressing the factors associated with the chronicity of the CHIKV infection [17]. Rheumatic lesions in the chronic phase of the CHIKF are believed to result from the virus’ ability to reproduce, and its ability to generate tissue damage in the joint, the severity of which varies according to the immune response of the infected individual [17,53,54–56].

The main risk factors for arthralgia in the chronic phase, according to the Brazilian Ministry of Health, are: patient over 45 years of age; pre-existing joint impairment; high-intensity joint injuries in the acute phase, the latter being the most common and persistent symptom, characterized by pain with or without the presence of edema, functional limitations, deformity and absence of erythema [1].

Regarding clinical management, studies have cited the use of disease-modifying antirheumatic drugs as being effective in treatment during the chronic phase of CHIKV arthritis with ongoing synovitis or tenosynovitis [33,57–60].

Regarding aggravating factors and comorbidities of the chronic phase, Brazilian Society of Rheumatology indicates that, in addition to routine examinations, the need for antibody research and synovial fluid analysis should be observed whenever clinical signs indicate any chronic joint disease, for it to assist in the differential diagnosis [17].

Apart from arthralgia, other signs and symptoms were observed in the chronic phase of CHIKF. The Brazilian Ministry of Health, and other studies mention, among other chronic manifestations resulting from the CHIKV infection, the following impairments: fatigue, cephalea, pruritus, alopecia, rash, dysesthesia, paresthesia, neuropathic pain, Raynaud’s phenomenon, cerebellar alterations, sleep disorders, memory alterations, attention deficit, mood alterations, blurred vision and depression [1,3,7,9].

In the present review, we observed the presence of chronic fatigue and depression among the affected individuals. These impairments can be interpreted as chronic inflammatory neuropsychiatric disorders that occur more frequently in epidemics, in addition to sleep and digestive disorders, among other signs and symptoms [61]. Such impairments are related to the increase in pro-inflammatory biomarkers that induce depression (cytokines) [62–64] and chronic fatigue (neopterins) [63,64].

Another factor to be observed is the fact that ten of the 29 studies included in this review come from Reunion Island (France), carried out between 2008 and 2018, demonstrating the great impact that CHIKV had on this island.

Our study presents some limitations. A limitation, common in systematic reviews, is that the reliability of the results is limited by the quality of the included studies. The presence of retrospective studies–which are more subject to difficulties in obtaining information of interest for the review–, and of different protocols for investigating clinical manifestations presented by each study should be taken into account.

It is also noteworthy that a selected study [40] has almost half of the total sample, which has a great influence on the results in relation to the sample size. However, this study presented only 89 individuals with arthralgia, which was the only manifestation evaluated.

The fact that it has some studies with a high risk of bias in its methodology, based on subjective evaluations self-reported by the patients, sometimes without any clinical examination, may generate overestimation and/or underestimation of the prevalence of chronic signs and symptoms. However, it is important to note that some rheumatological clinical manifestations, such as arthralgia and myalgia, can only be measured through self-assessment. Another limitation is the lack of socioeconomic data records, which is a pendency for further investigation.

## Final considerations

This systematic review sought to observe the prevalence of clinical signs and symptoms of the chronic phase of the Chikungunya Fever, and evidenced arthralgia as the main manifestation shown in this phase. Once arthralgia, myalgia, fatigue, sleep disorders, skin lesions, depression and digestive disorders is an often uneasy symptoms, it can affect the quality of life of stricken individuals, with implications in their social and work life. Since the chronic phase is common in infected individuals, all levels of health care should be prepared to monitor, in the medium to long term, patients affected by this condition.

Studies addressing the socioeconomic impact caused by the Chikungunya virus infection are suggested, to characterize how the chronicity of this condition affects the social and work life of affected individuals. The need for research seeking models of laboratory diagnostic prediction capable of optimizing the differentiation between this condition and other arboviruses is also reinforced, allowing an earlier clinical intervention.

## Supporting information

S1 Appendix Presents the assessment of the methodological quality of the studies included in this review, using the tool “Joanna Briggs Institute’s critical appraisal checklist for studies reporting prevalence data”. (DOCX)

S1 PRISMA Checklist Presents the criteria for carrying out this review in accordance with the PRISMA recommendation. (DOCX)
