# Supplementary material for: Clinical manifestations associated with the chronic phase of Chikungunya Fever: A systematic review of prevalence
Source: PLoS Negl Trop Dis. 2025 Feb 3;19(2):e0012810. doi: 10.1371/journal.pntd.0012810 (PMC11825093; doi:10.1371/journal.pntd.0012810)
Supplement: S1 Appendix — (DOCX) [file pntd.0012810.s001.docx]

S1 Appendix - risk of bias and methodological quality evaluated by the tool “Joanna Briggs Institute’s critical appraisal checklist for studies reporting prevalence data”

| Authors | Q1 | Q2 | Q3 | Q4 | Q5 | Q6 | Q7 | Q8 | Q9 | % yes / Risk of Bias |
| --- | --- | --- | --- | --- | --- | --- | --- | --- | --- | --- |
| BORGHERINI et al [1] | √ | √ | √ | √ | √ | √ | √ | √ | √ | 100% / Low |
| BOUQUILLARD, COMBE [2] | √ | √ | X | √ | √ | √ | √ | X | √ | 77,8% / Low |
| SISSOKO et al [3] | √ | √ | √ | √ | √ | X | X | √ | X | 66,7% / Moderate |
| SOUMAHORO et al [4] | √ | √ | √ | √ | √ | X | X | √ | √ | 77,8% / Low |
| LARRIEU et al [5] | √ | √ | X | X | √ | √ | X | X | X | 44,4% / High |
| MANIMUNDA et al [6] | √ | √ | √ | √ | √ | √ | √ | X | √ | 88,9% / Low |
| GERARDIN et al [7] | √ | √ | √ | √ | √ | √ | √ | √ | X | 88,9% / Low |
| MATHEW et al [8] | √ | X | √ | √ | √ | X | X | X | X | 44,4% / High |
| CHOPRA et al [9] | √ | √ | √ | √ | √ | √ | √ | √ | √ | 100% / Low |
| COUTURIER et al [10] | √ | √ | √ | √ | √ | √ | √ | √ | √ | 100% / Low |
| MORO et al [11] | √ | √ | √ | √ | √ | √ | √ | √ | √ | 100% / Low |
| ESSACKJEE et al [12] | √ | X | √ | √ | √ | X | X | √ | √ | 66,7% / Moderate |
| MOHD ZIM et al [13] | √ | √ | X | √ | √ | √ | √ | √ | √ | 88,9% / Low |
| SCHILTE et al [14] | √ | √ | X | X | √ | √ | √ | √ | X | 66,7% / Moderate |
| RAMACHANDRAN et al [15] | X | √ | X | X | √ | I | √ | √ | X | 44,4% / High |
| JAVELLE et al [16] | √ | √ | √ | X | √ | √ | √ | X | √ | 77,8% / Low |
| MARIMOUTOU et al [17] | √ | X | √ | X | √ | X | X | X | √ | 44,4% / High |
| van GENDEREN et al [18] | √ | X | √ | X | √ | X | X | √ | √ | 55,5% / Moderate |
| RODRIGUEZ-MORALES et al [19] | √ | √ | √ | √ | √ | √ | √ | √ | √ | 100% / Low |
| ZEANA et al [20] | √ | √ | X | √ | √ | √ | √ | √ | √ | 88,9% / Low |
| BOUQUILLARD et al [21] | √ | √ | √ | √ | √ | √ | √ | √ | √ | 100% / Low |
| CONSUEGRA-RODRIGUEZ et al [22] | √ | √ | X | X | √ | √ | X | √ | X | 55,5% / Moderate |
| CHANG et al [23] | √ | √ | √ | √ | √ | √ | X | √ | √ | 88,9% / Low |
| DUVIGNAUD et al [24] | √ | √ | √ | √ | √ | √ | √ | √ | √ | 100% / Low |
| HUITS et al [25] | √ | √ | √ | X | √ | √ | √ | √ | X | 77,8% / Low |
| MURILLO-ZAMORA et al [26] | √ | √ | √ | √ | √ | √ | √ | √ | √ | 100% / Low |
| PETERS et al [27] | √ | √ | X | √ | √ | √ | √ | √ | X | 77,8% / Low |
| de MORAES et al [28] | √ | √ | √ | √ | √ | √ | √ | √ | √ | 100% / Low |
| NINLA-AESONG et al [29] | √ | √ | √ | √ | √ | √ | √ | √ | √ | 100% / Low |

Note: √ - Yes; X - No; I – Uncertain;

Q1) Was the sample frame appropriate to address the target population? Q2) Were study participants sampled in an appropriate way? Q3) Was the sample size adequate? Q4) Were the study subjects and the setting described in detail? Q5) Was the data analysis conducted with sufficient coverage of the identified sample? Q6) Were valid methods used for the identification of the condition? Q7) Was the condition measured in a standard, reliable way for all participants? Q8) Was there appropriate statistical analysis? Q9) Was the response rate adequate, and if not, was the low response rate managed appropriately?

**REFERENCES**

1. Borgherini G, Poubeau P, Jossaume A, Gouix A, Cotte L, Michault A, et al. Persistent arthralgia associated with chikungunya virus: a study of 88 adult patients on reunion island. *Clin Infect Dis* 2008; 47:469e75. [http://dx.doi.org/10.1086/590003]

2. Bouquillard E, Combe B. A report of 21 cases of rheumatoid arthritis following Chikungunya fever. A mean follow-up of two years. *Joint Bone Spine.* 2009 Dec; 76(6):654-7.

3. Sissoko D, Malvy D, Ezzedine K, Renault P, Moscetti F, Ledrans M, et al. Postepidemic Chikungunya disease on reunion island: Course of rheumatic manifestations and associated factors over a 15-month period. *PLoS Negl Trop Dis* 2009; 3:1e6. [http://dx.doi.org/10.1371/journal.pntd.0000389]

4. Soumahoro M-K, Gerardin P, Boelle P-Y, Perrau J, Fianu A, Pouchot J, et al. Impact of Chikungunya virus infection on health status and quality of life: a retrospective cohort study. *PLoS One* 2009;4:e7800. [http://dx.doi.org/10.1371/journal.pone.0007800]

5. Larrieu S, Pouderoux N, Pistone T, Filleul L, Receveur MC, Sissoko D, et al. Factors associated with persistence of arthralgia among chikungunya virus-infected travellers: report of 42 French cases. *J Clin Virol* 2010; 47:85e8. [http://dx.doi.org/10.1016/j.jcv.2009.11.014]

6. Manimunda SP, Vijayachari P, Uppoor R, Sugunan AP, Singh SS, Rai SK, et al. Clinical progression of Chikungunya fever during acute and chronic arthritic stages and the changes in joint morphology as revealed by imaging. *Trans R Soc Trop Med Hyg* 2010; 104:392e9. [http://dx.doi.org/10.1016/j.trstmh.2010.01.011]

7. Gerardin P, Fianu A, Malvy D, Mussard C, Boussaïd K, Rollot O, et al. Perceived morbidity and community burden after a Chikungunya outbreak: the TELECHIK survey, a population-based cohort study. *BMC Med* 2011; 9:1e11. [http://dx.doi.org/10.1186/1741-7015-9-5]

8. Mathew AJ, Goyal V, George E, Thekkemuriyil DV, Jayakumar B, Chopra A. Rheumatic-musculoskeletal pain and disorders in a naïve group of individuals 15 months following a Chikungunya viral epidemic in south India: a population based observational study. *Int J Clin Pract* 2011; 65:1306e12. [http://dx.doi.org/10.1111/j.1742-1241.2011.02792.x]

9. Chopra A, Anuradha V, Ghorpade R, Saluja M. Acute Chikungunya and persistent musculoskeletal pain following the 2006 Indian epidemic: a 2-year prospective rural community study. *Epidemiol Infect* 2012; 140:842e50. [http://dx.doi.org/10.1017/S0950268811001300]

10. Couturier E, Guillemin F, Mura M, Leon L, Virion J-M, Letort M-J, et al. Impaired quality of life after chikungunya virus infection: a 2-year follow-up study. *Rheumatology* 2012; 51:1315e22. [http://dx.doi.org/10.1093/rheumatology/kes015]

11. Moro ML, Grilli E, Corvetta A, Silvi G, Angelini R, Mascella F, et al. Long-term chikungunya infection clinical manifestations after an outbreak in Italy: a prognostic cohort study. *J Infect* 2012; 65:165e72. [http://dx.doi.org/10.1016/j.jinf.2012.04.005]

12. Essackjee K, Goorah S, Ramchurn SK, Cheeneebash J, Walker-Bone K. Prevalence of and risk factors for chronic arthralgia and rheumatoid-like polyarthritis more than 2 years after infection with chikungunya virus. *Postgrad Med J* 2013; 89:440e7. [http://dx.doi.org/10.1136/postgradmedj-2012-131477]

13. Mohd Zim MA, Sam IC, Omar SFS, Chan YF, AbuBakar S, Kamarulzaman A. Chikungunya infection in Malaysia: Comparison with dengue infection in adults and predictors of persistent arthralgia. *J Clin Virol* 2013; 56:141e5. [http://dx.doi.org/10.1016/j.jcv.2012.10.019]

14. Schilte C, Staikovsky F, Couderc T, Madec Y, Carpentier F, Kassab S, et al. Chikungunya virus-associated long-term arthralgia: a 36-month prospective longitudinal study. *PLoS Negl Trop Dis* 2013:7. [http://dx.doi.org/10.1371/journal.pntd.0002137]

15. Ramachandran V, Kaur P, Kanagasabai K, Vadivoo S, Murhekar MV. Persistent arthralgia among Chikungunya patients and associated risk factors in Chennai, South India. *Postgrad Med J* 2014; 60:3e6. [http://dx.doi.org/10.4103/0022-3859.128795]

16. Javelle E, Ribera A, Degasne I, Gaüzère BA, Marimoutou C, Simon F. Specific Management of Post-Chikungunya Rheumatic Disorders: A Retrospective Study of 159 Cases in Reunion Island from 2006-2012. *PLoS Negl Trop Dis* 2015; 9:1e18. [http://dx.doi.org/10.1371/journal.pntd.0003603]

17. Marimoutou C, Ferraro J, Javelle E, Deparis X, Simon F. Chikungunya infection: self-reported rheumatic morbidity and impaired quality of life persist 6 years later. *Clin Microbiol Infect* 2015;21:688e93. [http://dx.doi.org/10.1016/j.cmi.2015.02.024]

18. van Genderen FT, Krishnadath I, Sno R, Grunberg MG, Zijlmans W, Adhin MR. First Chikungunya Outbreak in Suriname; Clinical and Epidemiological Features. *PLoS Negl Trop Dis* 2016;10(4):e0004625. doi: [10.1371/journal.pntd.0004625]

19. Rodríguez-Morales AJ, Calvache-Benavides CE, Giraldo-Gómez J, Hurtado-Hurtado N, Yepes-Echeverri MC, García-Loaiza CJ, et al. Post-chikungunya chronic arthralgia: results from a retrospective follow-up study of 131 cases in Tolima, Colombia, *Travel Medicine and Infectious Disease* (2015), doi: [10.1016/j.tmaid.2015.09.001]

20. Zeana C, Kelly P, Heredia W, Cifuentes A, Franchin G, Purswani M, et al. Postchikungunya rheumatic disorders in travelers after return from the Caribbean. *Travel Med Infect Dis* 2016; 14:21e5. [http://dx.doi.org/10.1016/j.tmaid.2016.01.009]

21. Bouquillard E, Fianu A, Bangil M, Charlette N, Ribera A, Michault A, et al. Rheumatic manifestations associated with Chikungunya virus infection: A study of 307 patients with 32-month follow-up (RHEUMATOCHIK study). *Joint Bone Spine* 2017 Feb; S1297-319X(17)30034-9.

22. Consuegra-Rodríguez MP, Hidalgo-Zambrano DM, Vásquez-Serna H, Jimenez-Canizales CE, Parra-Valencia E, Rodriguez-Morales AJ. Post-chikungunya chronic inflammatory rheumatism: Follow-up of cases after 1 year of infection in Tolima, Colombia. *Travel Med Infect Dis.* 2017 Dec; S1477-8939(17)30201-6.

23. Chang AY, Encinales L, Porras A, Pacheco N, Reid SP, Martins KAO, et al. Frequency of chronic joint pain following Chikungunya Virus infection. A Colombian cohort study. *Arthrits & Rheumatology* 2018. 70(4):578-584.

24. Duvignaud A, Fianu A, Bertolotti A, Jaubert J, Michault A, Poubeau P, et al. Rheumatism and chronic fatigue, the two facets of post-chikungunya disease: the TELECHIK cohort study on Reunion Island. *Epidemiol Infect.* 2018; 28:1-9. doi: [10.1017/S0950268818000031]

25. Huits R, De Kort J, Van Den Berg R, Chong L, Tsoumanis A, Eggermont K, et al. Chikungunya virus infection in Aruba: Diagnosis, clinical features and predictors of post-chikungunya chronic polyarthralgia. *PLoS ONE* 2018.13(4): e0196630. [https://doi.org/10.1371/journal.pone.0196630]

26. Murillo-Zamora E, Mendoza-Cano O, Trujillo-Hernández B, Guzmán-Esquivel J, Higareda-Almaraz E, Higareda-Almaraz MA, et al. Persistent arthralgia and related risks factors: A cohort study at 12 months from laboratory-confirmed Chikungunya infection. *Archives of Medical Research*. 2018.

27. Peters CMM, Pijnacker R, Fanoy EB, Bouwman LJT, de Langen LE, van den Kerkhof JHTC, et al. Chikungunya virus outbreak in Sint Maarten: Long-term arthralgia after a 15-month period. *J Vector Borne Dis 55*, Jun 2018:137–143.

28. de Moraes L, Cerqueira-Silva T, Nobrega V, Akrami K, Santos LA, Orge C, et al. A clinical scoring system to predict long-term arthralgia in Chikungunya disease: A cohort study. *PLoS Negl Trop Dis* 2020.14(7):e0008467. [https://doi.org/10.1371/journal.pntd.0008467]

29. Ninla-aesong P, Mitamun W, Noipha K. Long-term persistence of Chikungunya Virus-associated manifestations and Anti-Chikungunya Virus antibody in southern Thailand: 5 years after an outbreak in 2008-2009. *Viral Immunology* 2020. 33(2):1-8.
