# Supplementary material for: Clinical manifestations associated with the chronic phase of Chikungunya Fever: A systematic review of prevalence
Source: PLoS Negl Trop Dis. 2025 Feb 3;19(2):e0012810. doi: 10.1371/journal.pntd.0012810 (PMC11825093; doi:10.1371/journal.pntd.0012810)
Supplement: S1 PRISMA Checklist — (DOCX) [file pntd.0012810.s002.docx]

# PRISMA 2020 Main Checklist

| **Topic** | **No.** | **Item** | **Location where item is reported** |
| --- | --- | --- | --- |
| **TITLE** |  |  |  |
| **Title** | 1 | Identify the report as a systematic review. | CLINICAL MANIFESTATIONS ASSOCIATED WITH THE CHRONIC PHASE OF CHIKUNGUNYA FEVER: A SYSTEMATIC REVIEW OF PREVALENCE |
| **ABSTRACT** |  |  |  |
| **Abstract** | 2 | See the PRISMA 2020 for Abstracts checklist |  |
| **INTRODUCTION** |  |  |  |
| **Rationale** | 3 | Describe the rationale for the review in the context of existing knowledge. | The knowledge acquired regarding the high prevalence of several arboviruses present in the world, some of which spread rapidly to various regions of the planet, generating coexistence and coinfections, demonstrates the importance of a better understanding of the impact that each of them has on the health of infected individuals. In this scenario, differential diagnosis becomes an essential factor for the correct treatment of the signs and symptoms presented. Therefore, with this study we sought to expand knowledge about the epidemiology and clinical manifestations present in arboviruses, highlighting the Chikungunya Virus as one of the most prevalent and symptomatic arboviruses. |
| **Objectives** | 4 | Provide an explicit statement of the objective(s) or question(s) the review addresses. | We conducted a systematic review of prevalence, with the objective of describing the clinical manifestations of the Chikungunya Virus disease in its chronic phase. |
| **METHODS** |  |  |  |
| **Eligibility criteria** | 5 | Specify the inclusion and exclusion criteria for the review and how studies were grouped for the syntheses. | The inclusion criteria for the studies were: tracking the individuals in the cohort, infected by Chikungunya Virus via vector transmission and with laboratory confirmation using reverse transcription, followed by polymerase chain reaction (RT-PCR) for at least six months; describing the clinical manifestations presented in the chronic phase of Chikungunya Fever. As exclusion criteria we have: different studies, but from the same region, that addressed the same cohort of individuals. Potentially relevant articles were individually evaluated by two reviewers. A flow diagram was created, which describes the study selection process, based on the PRISMA Recommendation (Preferred Reporting Items for Systematic Reviews and Meta-Analyses), used for this study's design. Disagreements regarding the inclusion and exclusion of studies among the reviewers were solved through the evaluation and opinion of a third reviewer. The search for the articles was closed in October 12, 2024. |
| **Information sources** | 6 | Specify all databases, registers, websites, organisations, reference lists and other sources searched or consulted to identify studies. Specify the date when each source was last searched or consulted. | The research was conducted using the electronic databases MEDLINE (accessed via PubMed) and EMBASE. The search for the articles was closed in October 12, 2024. |
| **Search strategy** | 7 | Present the full search strategies for all databases, registers and websites, including any filters and limits used. | The following keywords were used: Chikungunya Virus; Chikungunya Fever; Arthralgia, Fever, Signs and Symptoms, using Boolean operators OR and AND to associate these variables as a research strategy - ((“Chikungunya Virus” OR “Chikungunya Fever”) AND (Arthralgia OR Fever OR “Signs and Symptoms”)). No limits were applied for language or year of publication. |
| **Selection process** | 8 | Specify the methods used to decide whether a study met the inclusion criteria of the review, including how many reviewers screened each record and each report retrieved, whether they worked independently, and if applicable, details of automation tools used in the process. | Potentially relevant articles were individually evaluated by two reviewers. Disagreements regarding the inclusion and exclusion of studies among the reviewers were solved through the evaluation and opinion of a third reviewer. |
| **Data collection process** | 9 | Specify the methods used to collect data from reports, including how many reviewers collected data from each report, whether they worked independently, any processes for obtaining or confirming data from study investigators, and if applicable, details of automation tools used in the process. | The analysis of the information from the included studies was based on: the description of the study’s design (prospective or retrospective); the number of participants included in each study, as well as the total number of individuals included in this review; the mean age; the length of each cohort's follow-up; the presence of signs and symptoms observed in the chronic phase of Chikungunya Fever; the observation of the presence or absence of factors concomitant to the chronic phase and/or comorbidities. |
| **Data items** | 10a | List and define all outcomes for which data were sought. Specify whether all results that were compatible with each outcome domain in each study were sought (e.g. for all measures, time points, analyses), and if not, the methods used to decide which results to collect. | The analysis of the information from the included studies was based on: the description of the study’s design (prospective or retrospective); the number of participants included in each study, as well as the total number of individuals included in this review; the mean age; the length of each cohort's follow-up; the presence of signs and symptoms observed in the chronic phase of CHIKF; the observation of the presence or absence of factors concomitant to the chronic phase and/or comorbidities. |
|  | 10b | List and define all other variables for which data were sought (e.g. participant and intervention characteristics, funding sources). Describe any assumptions made about any missing or unclear information. | Not applicable. |
| **Study risk of bias assessment** | 11 | Specify the methods used to assess risk of bias in the included studies, including details of the tool(s) used, how many reviewers assessed each study and whether they worked independently, and if applicable, details of automation tools used in the process. | The risk of bias in the selected studies was assessed using the “Joanna Briggs Institute's critical appraisal checklist for studies reporting prevalence data”, a revised tool that was specifically developed for researches that present prevalence data. Each research included in this study was categorized according to the percentage of affirmative answers among the nine questions available in the evaluation tool. The risk of bias was considered high when the study presented up to 49% of the answers classified as "yes", moderate when obtained from 50% to 69%, and low when the survey exceeded 70% of "yes" scores. |
| **Effect measures** | 12 | Specify for each outcome the effect measure(s) (e.g. risk ratio, mean difference) used in the synthesis or presentation of results. | Study results were presented using simple descriptive statistics, such as absolute values ​​and means. |
| **Synthesis methods** | 13a | Describe the processes used to decide which studies were eligible for each synthesis (e.g. tabulating the study intervention characteristics and comparing against the planned groups for each synthesis (item 5)). | All studies included in this review were used for the synthesis. |
|  | 13b | Describe any methods required to prepare the data for presentation or synthesis, such as handling of missing summary statistics, or data conversions. | Study results were presented using simple descriptive statistics, such as absolute values ​​and means. No additional methods were required. |
|  | 13c | Describe any methods used to tabulate or visually display results of individual studies and syntheses. | The results of the review were presented using a flowchart and tables. |
|  | 13d | Describe any methods used to synthesize results and provide a rationale for the choice(s). If meta-analysis was performed, describe the model(s), method(s) to identify the presence and extent of statistical heterogeneity, and software package(s) used. | The results were presented in a flowchart (following the PRISMA model) and in descriptive tables in order to provide the information in a clear and objective manner. |
|  | 13e | Describe any methods used to explore possible causes of heterogeneity among study results (e.g. subgroup analysis, meta-regression). | Not applicable |
|  | 13f | Describe any sensitivity analyses conducted to assess robustness of the synthesized results. | Not applicable |
| **Reporting bias assessment** | 14 | Describe any methods used to assess risk of bias due to missing results in a synthesis (arising from reporting biases). | Not applicable |
| **Certainty assessment** | 15 | Describe any methods used to assess certainty (or confidence) in the body of evidence for an outcome. | Not applicable |
| **RESULTS** |  |  |  |
| **Study selection** | 16a | Describe the results of the search and selection process, from the number of records identified in the search to the number of studies included in the review, ideally using a flow diagram. | The search identified 175 articles, which underwent abstract screening and subsequent eligibility according to the inclusion criteria, defining the process that totaled 29 studies selected to compose this review). The 109 studies that were excluded after analysis of the abstract did not present an observational design. The 37 articles excluded after analysis of the full text did not present a minimum follow-up time of six months or did not address the chronic phase of the disease. A flowchart was developed, included in the study. |
|  | 16b | Cite studies that might appear to meet the inclusion criteria, but which were excluded, and explain why they were excluded. | Bower et al. (2021) conducted a cohort study with patients infected with the Chikungunya virus, evaluating their signs and symptoms over time. However, their cohort did not meet the minimum of six months adopted in our review as an inclusion criterion. Therefore, this study was excluded from our review. Bower H, el Karsany M, Adam AAAH, Idriss MI, Alzain MA, Alfakiyousif MEA, et al. (2021) “Kankasha” in Kassala: A prospective observational cohort study of the clinical characteristics, epidemiology, genetic origin, and chronic impact of the 2018 epidemic of Chikungunya virus infection in Kassala, Sudan. PLoS Negl Trop Dis 15(4): e0009387. https://doi. org/10.1371/journal.pntd.0009387.  Similarly, the study by Segura-Charry et al. (2021), which evaluated musculoskeletal disorders in patients who presented Chikungunya Fever, performed a retrospective cohort in patients with recurrent symptoms for only 3 months, and was also excluded for this reason. Juan Sebastián Segura-Charry, Maria Alexandra Parada-Martinez, |
| **Study characteristics** | 17 | Cite each included study and present its characteristics. | All studies included in this review are observational studies that evaluated cohorts (prospective or retrospective) of patients infected with the Chikungunya virus who presented chronic signs and symptoms related to the disease six months or more after diagnosis. The studies were conducted in several countries, present in Latin America, North America, Africa, Asia and Europe. |
| **Risk of bias in studies** | 18 | Present assessments of risk of bias for each included study. | Studies were assessed using the tool “Joanna Briggs Institute’s critical appraisal checklist for studies reporting prevalence data”. Available in Appendix A. |
| **Results of individual studies** | 19 | For all outcomes, present, for each study: (a) summary statistics for each group (where appropriate) and (b) an effect estimate and its precision (e.g. confidence/credible interval), ideally using structured tables or plots. | The results of each study were presented clearly and objectively using tables, which are included in this review. |
| **Results of syntheses** | 20a | For each synthesis, briefly summarise the characteristics and risk of bias among contributing studies. | Studies were assessed using the tool “Joanna Briggs Institute’s critical appraisal checklist for studies reporting prevalence data”. Available in Appendix A. |
|  | 20b | Present results of all statistical syntheses conducted. If meta-analysis was done, present for each the summary estimate and its precision (e.g. confidence/credible interval) and measures of statistical heterogeneity. If comparing groups, describe the direction of the effect. | The characteristics of the included studies are described in table 1. Among the 29 studies, 18 presented retrospective design, and 11 presented prospective design. The study with the smallest cohort of individuals used 21 subjects, and the largest used 5,344 subjects. The total sample consists of 11,378 individuals. The mean age ranged from 32 to 58.3 years. The minimum follow-up time observed was of 6 months, while the longest follow-up was of 72 months. The results derived from signs and symptoms observed in the chronic phase of Chikungunya Fever in the studies used are shown in table 2. Among the 29 studies included in this review, only one did not identify arthralgia as a prevalent symptom in the chronic phase of Chikungunya Fever. Other signs and symptoms observed in the selected studies were: fatigue; myalgia; sleep disorders; skin lesions; depression; and digestive disorders. |
|  | 20c | Present results of all investigations of possible causes of heterogeneity among study results. | In order to obtain the most reliable prevalence data regarding chronic signs and symptoms of Chikungunya Fever, we sought to ensure that all studies included in this review presented methodologically similar designs. |
|  | 20d | Present results of all sensitivity analyses conducted to assess the robustness of the synthesized results. | Not applicable |
| **Reporting biases** | 21 | Present assessments of risk of bias due to missing results (arising from reporting biases) for each synthesis assessed. | Not applicable |
| **Certainty of evidence** | 22 | Present assessments of certainty (or confidence) in the body of evidence for each outcome assessed. | Not Applicable |
| **DISCUSSION** |  |  |  |
| **Discussion** | 23a | Provide a general interpretation of the results in the context of other evidence. | This systematic review sought to observe the prevalence of clinical signs and symptoms of the chronic phase of Chikungunya Fever, and highlighted arthralgia as the main manifestation presented in this phase. The results of chronic arthralgia in patients from Latin America contained in this review corroborated another study that performed the same prevalence analysis in this region. Since arthralgia, myalgia, fatigue, sleep disorders, skin lesions, depression and digestive disorders are frequently bothersome symptoms, also reported in other studies, they can affect the quality of life of affected individuals, with implications for their social and professional life. |
|  | 23b | Discuss any limitations of the evidence included in the review. | Our study presents some limitations. A limitation, common in systematic reviews, is that the reliability of the results is limited by the quality of the included studies. The presence of retrospective studies – which are more subject to difficulties in obtaining information of interest for the review –, and of different protocols for investigating clinical manifestations presented by each study should be taken into account. It is also noteworthy that a selected study has almost half of the total sample, which has a great influence on the results in relation to the sample size. However, this study presented only 89 individuals with arthralgia, which was the only manifestation evaluated. The fact that it has some studies with a high risk of bias in its methodology, based on subjective evaluations self-reported by the patients, sometimes without any clinical examination, may generate overestimation and/or underestimation of the prevalence of chronic signs and symptoms. However, it is important to note that so |
|  | 23c | Discuss any limitations of the review processes used. | This study did not have any limitations in the review process used. |
|  | 23d | Discuss implications of the results for practice, policy, and future research. | Since the chronic phase is common in infected individuals, all levels of health care should be prepared to monitor, in the medium to long term, patients affected by this condition. Studies addressing the socioeconomic impact caused by the Chikungunya virus infection are suggested, to characterize how the chronicity of this condition affects the social and work life of affected individuals. The need for research seeking models of laboratory diagnostic prediction capable of optimizing the differentiation between this condition and other arboviruses is also reinforced, allowing an earlier clinical intervention. |
| **OTHER INFORMATION** |  |  |  |
| **Registration and protocol** | 24a | Provide registration information for the review, including register name and registration number, or state that the review was not registered. | This review has not been registered. |
|  | 24b | Indicate where the review protocol can be accessed, or state that a protocol was not prepared. | This review has not been registered. |
|  | 24c | Describe and explain any amendments to information provided at registration or in the protocol. | Not applicable. |
| **Support** | 25 | Describe sources of financial or non-financial support for the review, and the role of the funders or sponsors in the review. | The authors received no specific funding for this work. |
| **Competing interests** | 26 | Declare any competing interests of review authors. | The authors have declared that no competing interests exist. |
| **Availability of data, code and other materials** | 27 | Report which of the following are publicly available and where they can be found: template data collection forms; data extracted from included studies; data used for all analyses; analytic code; any other materials used in the review. | No items are publicly available at this time. |

#####

# PRIMSA Abstract Checklist

| **Topic** | **No.** | **Item** | **Reported?** |
| --- | --- | --- | --- |
| **TITLE** |  |  |  |
| **Title** | 1 | Identify the report as a systematic review. | Yes |
| **BACKGROUND** |  |  |  |
| **Objectives** | 2 | Provide an explicit statement of the main objective(s) or question(s) the review addresses. | Yes |
| **METHODS** |  |  |  |
| **Eligibility criteria** | 3 | Specify the inclusion and exclusion criteria for the review. | Yes |
| **Information sources** | 4 | Specify the information sources (e.g. databases, registers) used to identify studies and the date when each was last searched. | Yes |
| **Risk of bias** | 5 | Specify the methods used to assess risk of bias in the included studies. | Yes |
| **Synthesis of results** | 6 | Specify the methods used to present and synthesize results. | Yes |
| **RESULTS** |  |  |  |
| **Included studies** | 7 | Give the total number of included studies and participants and summarise relevant characteristics of studies. | Yes |
| **Synthesis of results** | 8 | Present results for main outcomes, preferably indicating the number of included studies and participants for each. If meta-analysis was done, report the summary estimate and confidence/credible interval. If comparing groups, indicate the direction of the effect (i.e. which group is favoured). | Yes |
| **DISCUSSION** |  |  |  |
| **Limitations of evidence** | 9 | Provide a brief summary of the limitations of the evidence included in the review (e.g. study risk of bias, inconsistency and imprecision). | No |
| **Interpretation** | 10 | Provide a general interpretation of the results and important implications. | Yes |
| **OTHER** |  |  |  |
| **Funding** | 11 | Specify the primary source of funding for the review. | No |
| **Registration** | 12 | Provide the register name and registration number. | No |

*From:* Page MJ, McKenzie JE, Bossuyt PM, Boutron I, Hoffmann TC, Mulrow CD, et al. The PRISMA 2020 statement: an updated guideline for reporting systematic reviews. MetaArXiv. 2020, September 14. DOI: 10.31222/osf.io/v7gm2. For more information, visit: [www.prisma-statement.org](C:\\Users\\Anna\\Downloads\\www.prisma-statement.org)
